# Activation of lateral hypothalamus-projecting parabrachial neurons by intraorally delivered gustatory stimuli

**DOI:** 10.3389/fncir.2014.00086

**Published:** 2014-07-29

**Authors:** Kenichi Tokita, William E. Armstrong, Steven J. St. John, John D. Boughter Jr.

**Affiliations:** ^1^Department of Anatomy and Neurobiology, University of Tennessee Health Science CenterMemphis, TN, USA; ^2^Department of Psychology, Rollins CollegeWinter Park, FL, USA

**Keywords:** taste, ingestion, parabrachial, hypothalamus, immunohistology

## Abstract

The present study investigated a subpopulation of neurons in the mouse parabrachial nucleus (PbN), a gustatory and visceral relay area in the brainstem, that project to the lateral hypothalamus (LH). We made injections of the retrograde tracer Fluorogold (FG) into LH, resulting in fluorescent labeling of neurons located in different regions of the PbN. Mice were stimulated through an intraoral cannula with one of seven different taste stimuli, and PbN sections were processed for immunohistochemical detection of the immediate early gene c-Fos, which labels activated neurons. LH projection neurons were found in all PbN subnuclei, but in greater concentration in lateral subnuclei, including the dorsal lateral subnucleus (dl). Fos-like immunoreactivity (FLI) was observed in the PbN in a stimulus-dependent pattern, with the greatest differentiation between intraoral stimulation with sweet (0.5 M sucrose) and bitter (0.003 M quinine) compounds. In particular, sweet and umami-tasting stimuli evoked robust FLI in cells in the dl, whereas quinine evoked almost no FLI in cells in this subnucleus. Double-labeled cells were also found in the greatest quantity in the dl. Overall, these results support the hypothesis that the dl contains direct a projection to the LH that is activated preferentially by appetitive compounds; this projection may be mediated by taste and/or postingestive mechanisms.

## Introduction

In rodents, the parabrachial nucleus of the pons (PbN) is a critical brainstem relay for both gustatory and visceral sensory information (for reviews, see Lundy and Norgren, 2004a; Saper, 2004; Sewards, 2004). Although physiological approaches in both rats and mice indicate general locations within PbN subnuclei for gustatory neurons (e.g., Halsell and Travers, 1997; Geran and Travers, 2009; Tokita and Boughter, 2012; Tokita et al., 2012), anterograde tracing studies in rats demonstrate there is gustatory and visceral overlap in most, if not all areas (Karimnamazi et al., 2002). In fact, studies have shown that the activity of PbN gustatory neurons can be modulated directly by gut manipulations (e.g., Baird et al., 2001).

From the PbN, both gustatory and visceral sensory information are relayed to cortex via the thalamus; other PbN neurons project directly to limbic forebrain areas, including the central nucleus of the amygdala (CeA), bed nucleus of the stria terminalis (BNST), and lateral hypothalamus (LH; Norgren, 1974; Saper and Loewy, 1980; Fulwiler and Saper, 1984; Halsell, 1992; Karimnamazi and Travers, 1998; Yamamoto, 2006; Tokita et al., 2010). The LH is involved in many aspects of feeding and intake (e.g., Hatton and Armstrong, 1981; Sawchenko, 1998; Simerly, 2004). Anatomically, PbN efferents terminate in the LH (Norgren, 1976; Saper and Loewy, 1980; Fulwiler and Saper, 1984; Bester et al., 1997; Bianchi et al., 1998; Karimnamazi and Travers, 1998; Yoshida et al., 2006; Tokita et al., 2010), and have been found to directly innervate orexin-containing neurons in this region (Niu et al., 2010). In turn, LH neurons project back to PbN (Moga et al., 1990; Saggu and Lundy, 2008; Tokita et al., 2009).

Gustatory-evoked responses have been directly recorded in the LH (Norgren, 1970; Yamamoto et al., 1989; Li et al., 2013). A direct projection of taste-responsive PbN neurons to LH was demonstrated via antidromic activation (Li et al., 2005). On the other hand, descending input from the LH has been shown to directly modulate PbN taste responses; both excitatory and inhibitory effects have been found (Lundy and Norgren, 2004b; Li et al., 2005; Lei et al., 2008). Lesion/behavior studies suggest that in addition to classically (and more recently) described effects on feeding and body weight (for review see Elmquist et al., 1999), the LH may play a role in hedonic assessment of taste stimuli in intake or taste reactivity tests (O’Kelly and Hatton, 1969; Vasudev et al., 1985; Ferssiwi et al., 1987; Touzani and Velley, 1990).

In this experiment, we tested the hypothesis that orally delivered taste stimuli that elicit appetitive behaviors preferentially stimulate LH-projecting PbN neurons in mice. This hypothesis was developed based on the fact that intraoral delivery of sweet and mildly salty-tasting stimuli, but not bitter-tasting stimuli, were shown to provoke robust expression of the immediate early gene c-Fos in regions of the PbN in rats that overlap with the location of clusters of LH-projection neurons (e.g., Fulwiler and Saper, 1984; Yamamoto et al., 1994; Yamamoto and Sawa, 2000a). We used a combination of retrograde tracing and intraoral-elicited c-Fos expression to examine potential subsets of activated neurons that directly project to the LH. In addition to sweet, bitter, and salty stimuli, we also used several umami stimuli, including monosodium glutamate (MSG), inosine 5’-monophosphate (IMP), and a mixture of these two stimuli. We have previously shown that such so-called “synergistic” mixtures provoke a robust, sweet-like response in PbN taste neurons in mice (Tokita and Boughter, 2012; Tokita et al., 2012).

## Materials and methods

### Subjects

A total of 35 male and female C57BL6/6J mice (22–29 g) were used. The animals were maintained in a temperature- and humidity-controlled colony room on a 12 h light/12 h dark cycle (lights on at 07:00 h, off at 19:00 h), and were given access to normal dry pellets (22/5 rodent diet, Harlan Teklad, Madison, WI, USA) and water. Food and water were available ad libitum except on the testing day and the previous day (see below). Sixteen additional mice were excluded from the final number due to unsuccessful tracer injections (i.e., one or the other injection not expelled properly, or not precisely on target). This study was approved by the Animal Care and Use Committee at UTHSC, and all experiments were carried out in accordance with the National Institute of Health Guide for Care and Use of Laboratory Animals (NIH Publications No. 80–23), revised 1996.

### Surgery

Mice were anesthetized with intraperitoneal injection of ketamine/xylazine (100/10 ml/kg; Fort Dodge Animal Health, Fort Dodge, IA, USA and Lloyd Laboratories, Shenandoah, IA, USA) and positioned in a stereotaxic frame (Stoelting, Wood Dale, IL, USA). The scalp was opened with a midline incision, and the skull was leveled between bregma and lambda by adjusting the bite bar. The body temperature was maintained at 35°C using a heating pad (Elenco electronics, Wheeling, IL, USA). Relative to bregma, a glass micropipette (25–30 μm tip diameter) filled with 5% fluorogold (FG) (Fluorochrome, Denver, CO, USA) was lowered into the LH (anteroposterior = −1.2, mediolateral = 1.2, dorsoventral = −5.2) with a micromanipulator (SM-191, Narishige, Tokyo, Japan). FG was injected into the right LH by iontophoresis (2 mA, 5 s on/off for 15 min) using a precision constant current source (Stoelting, Wood Dale, IL, USA). The position of the injection pipette was not altered 10 min prior to and 10 min following FG injection.

Following tracer injection, polyethylene tubing (PE 50) was inserted (with the aid of a 25 g needle) through the right buccal mucosa and led along the lateral surface of the skull. The needle was removed and the resulting cannula was secured to the skull using dental acrylic (Unifast Trad; GC Corporation, Tokyo, Japan) and screws (No. 19010-10; Fine Scientific Tools, Foster City, CA, USA). The oral end of the cannula was placed between the cheek and gum next to the first maxillary molar and flattened by flaring to prevent it from being drawn into the oral mucosa. These intraoral cannulas were inserted unilaterally, ipsilateral to the FG injection.

### Intraoral stimulation

Three days after surgery mice underwent an adaptation procedure, receiving distilled water through the intraoral cannula (0.1 ml/min for 15 min) using a precision syringe pump (model 341A; Sage Instruments, Cambridge, MA, USA) in a round Plexiglas test chamber (20 cm diameter, 14.5 cm height). This training occurred at roughly the same time (between 13:00 h and 15:00 h) for three consecutive days prior to the test day. On the last adaptation day mice were food and water restricted 20 h prior to testing, to promote sampling of stimuli.

On the test day, mice were placed in the test chamber at the same time of day as the adaptation procedure. For the next 15 min, mice were intraorally infused with 1.5 ml of one of the following stimuli using the same methods and rate used in the adaptation procedure: distilled water (*n* = 5), 0.5 M sucrose (*n* = 5), 0.1 M NaCl (*n* = 4), 0.003 M QHCl (*n* = 5), 0.1 M MSG (*n* = 5), 0.01 M IMP (*n* = 6), or a mixture of 0.1 M MSG and 0.01 M IMP (*n* = 5). Concentrations were selected on the basis of previous studies in rats (e.g., Yamamoto et al., 1994), as well as on our electrophysiological and behavioral studies in mice (e.g., Tokita et al., 2012 and unpublished studies). Following testing, mice were returned to home cages and given no additional food or water before perfusion.

### Perfusion and immunohistochemical staining

Two hours after the onset of the taste stimulation, the mice were anesthetized with 25% urethane (0.5 ml) and perfused transcardially with 0.02 M phosphate-buffered physiological saline (PBS) followed by ice-cold 4% paraformaldehyde in 0.1 M phosphate buffer. The brains were removed and postfixed in 4% paraformaldehyde for 1 day, and then transferred to a 30% buffered sucrose solution for cryoprotection and stored at 4°C for at least 1 week. Coronal sections (40 μm) of the PbN and LH were cut serially using a freezing microtome and divided into two adjacent series. One series was Nissl-stained with cresyl violet to reveal cytoarchitecture, and the adjacent series was used for examination of the fluorescent FG injection site, FG retrograde labeling and Fos-like immunoreactivity (FLI).

After rinsing in phosphate-buffered saline (PBS), the sections used for FLI were incubated in PBS containing 3% normal goat serum (NGS; Jackson ImmunoResearch Laboratories, West Grove, PA, USA) and 0.5% triton X-100 (Sigma-Aldrich, St. Louis, MO, USA) for 30 min. The sections were then incubated with a rabbit polyclonal anti-c-Fos antibody (sc-52, Santa Cruz Biotechnology, CA, USA) diluted to 1:5000 in PBS containing 3% NGS and 0.5% triton X-100 for 48 h at 4°C. Several sections were placed in the same solution only lacking the primary antibody to serve as a negative control. After rinsing in PBS, sections were soaked in 3% NGS in PBS for 30 min, and then incubated with a biotinylated goat anti-rabbit antibody (Jackson ImmunoResearch Laboratories, West Grove, PA, USA) diluted to 1:1000 in PBS containing 3% NGS and 0.5% triton X-100 for 120 min. The sections were then transferred to streptavidin–Cy3 (Jackson ImmunoResearch Laboratories, West Grove, PA, USA) or streptavidin–Alexa Fluor 568 (Molecular Probes/Invitrogen, San Diego, CA, USA) diluted to a concentration of 1:1000 in PBS. We found that this method resulted in less background staining than with a fluorescent secondary antibody (unpublished observations). No FLI was observed in observed in control sections.

Both cresyl violet and FLI series were mounted on silane-coated slides (Scientific Device Laboratory, Des Plaines, IL, USA), and coverslipped with mounting medium DPX (Fluka, Milwaukee, WI, USA) for bright field microscopy or Vectashield (Vector Laboratories, Burlingame, CA, USA) for fluorescence microscopy.

### Microscopic analysis of sections

Fluorescent labeling in all sections was imaged and analyzed using a Leica (DMRXA2, Leica Microsystems, Bannockburn, IL, USA) epifluorescence microscope equipped with a digital camera (Hamamatsu ORCA-ER, Hamamatsu Photonics, Shizuoka, Japan) and imaging software (SimplePCI, Hamamatsu Photonics, Shizuoka, Japan). FLI-positive cells were visualized with a rhodamine filter and FG with a DAPI filter. Single-labeled and double-labeled neurons were plotted and quantified from high-resolution digital microscopic images using ImageJ (National Institutes of Health, USA). In addition, a few sections from a single case were imaged using a confocal microscope (Zeiss 710, Carl Zeiss, Thornwood, NY) to show high-magnification examples of co-labeling (e.g., Figure 2).

**Figure 1:**
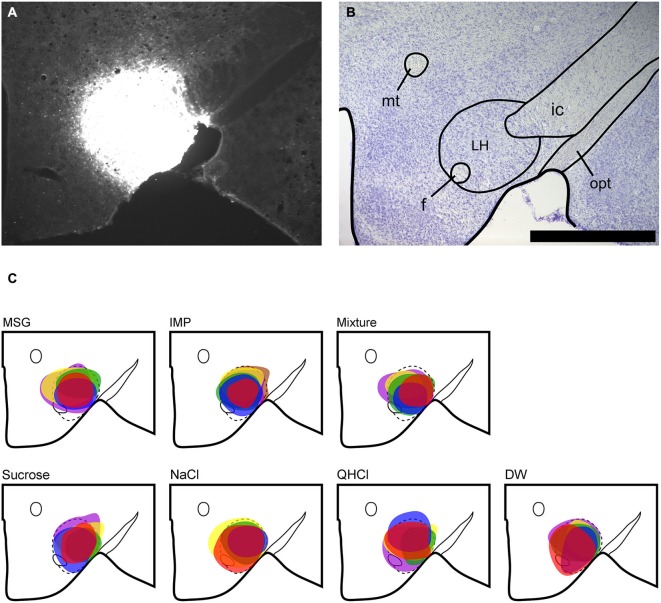
**LH injection sites. (A)** Representative fluorescent image of FG injection site in the LH. **(B)** Nissl-stained section showing level of LH injection. **(C)** Plots of injection site and size (maximal extent) for each animal according to stimulus group; note that colors only identify separate animals within each stimulus subgroup, and do not convey any meaning across groups. Scale bar = 500 μm. mt, mammillothalamic tract; f, fornix; ic, internal capsule; opt, optic tract.

**Figure 2:**
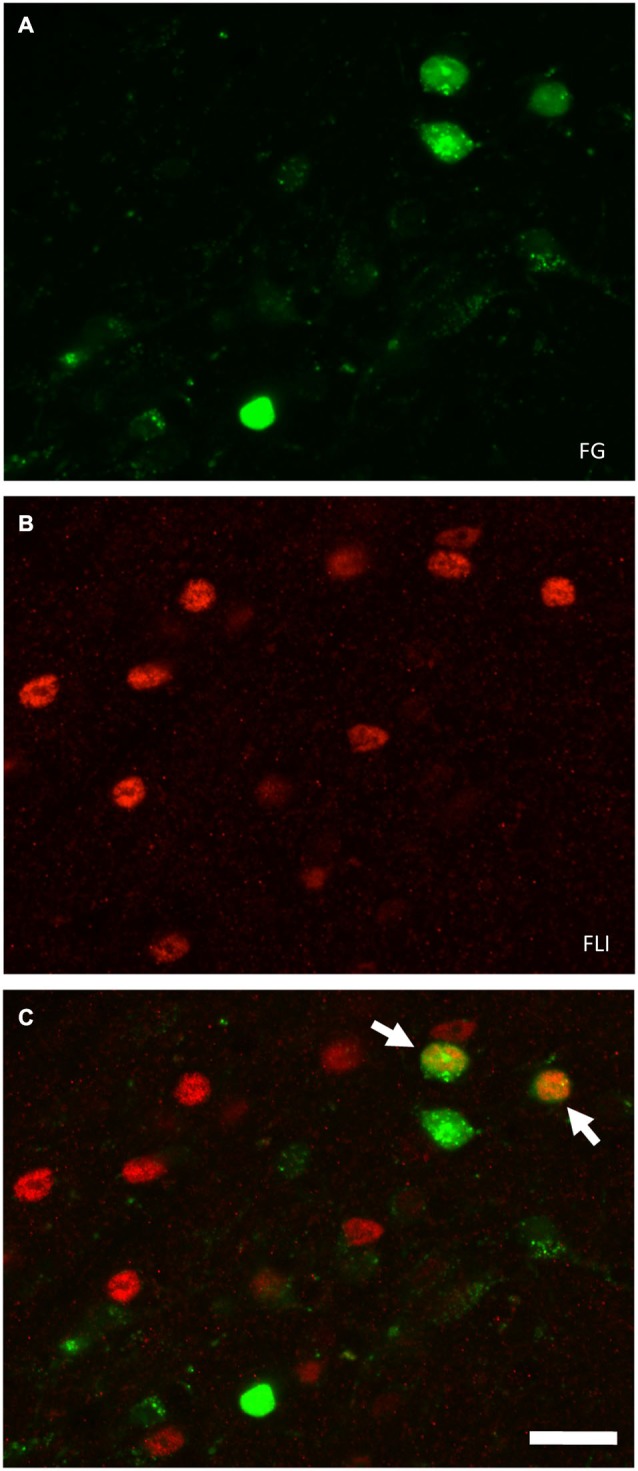
**Fluorescent images of double-labeled and single-labeled neurons in the ipsilateral PbN (dl subnucleus) following LH injections of FG**. Images were captured using a confocal microscope (Zeiss 710, Carl Zeiss MicroImagining, Inc., Thornwood, NY, USA). **(A)** FG-labeled cell somata. **(B)** FLI-positive nuclei. **(C)** In the merged image, there are two examples of double-labeled cells (arrows). Scale bar = 20 μm.

Delineation of seven PbN subnuclei was made with the use of the cresyl violet-stained sections, and according to previous studies in the mouse (Hashimoto et al., 2009; Tokita et al., 2010). Consistent with these prior studies, we did not subdivide the medial subnucleus into central medial and ventral medial subnuclei, a further delineation that has been made in studies with larger rodents (e.g., Halsell and Travers, 1997; Tokita et al., 2007), as it is difficult to make this distinction in mice

Both FG and FLI labeling in the PbN was examined in every other section along the rostral-caudal axis from a point about 200 μm rostral to the dorsal junction of the superior cerebellar peduncle (scp) with the mesencephalic trigeminal tract, to (most caudal) the initial appearance of the ventral lateral subnucleus (vl). This 500 μm stretch comprised an average of 7 fluorescently-stained sections in all mice, and likely captured the majority of gustatory-related regions in the mouse as defined by location of taste-responsive neurons from *in vivo* recording, FLI evoked by licking of 0.1 M NaCl, or density of cells projecting to the gustatory thalamus (Hashimoto et al., 2009; Tokita et al., 2010, 2012). Quantification of cells was made only on the side ipsilateral to the injection site and intraoral cannula. Although there are some PbN neurons that project to the contralateral LH, the majority project ipsilaterally (Tokita et al., 2010).

### Data analysis

For analysis of variance (ANOVA), assumptions of normality and homogeneity of variance were first assessed using the Kolmogorov-Smirnov and Bartlett’s test, respectively. FG and FLI cell counts (but not double-labeled cell counts) passed the great majority of these tests, and were analyzed with parametric statistics. For the PbN, mean counts of FG and FLI cells were initially compared between stimulus groups via within subjects (repeated measures) ANOVA (subdivision × stimulus). With significant variation in counts across subnucleus established, numbers of labeled neurons were compared across stimulus in each subnucleus with a one-way ANOVA followed by *post-hoc* comparison (Bonferroni multiple comparison test; *p* < 0.05). Double-labeled cells were analyzed with a non-parametric Kruskal-Wallis test, followed by multiple comparisons with Dunn’s test (*p* < 0.05). Mean proportions of double-labeled cells were compared between stimuli with a Mann-Whitney test.

## Results

**Figure 3:**
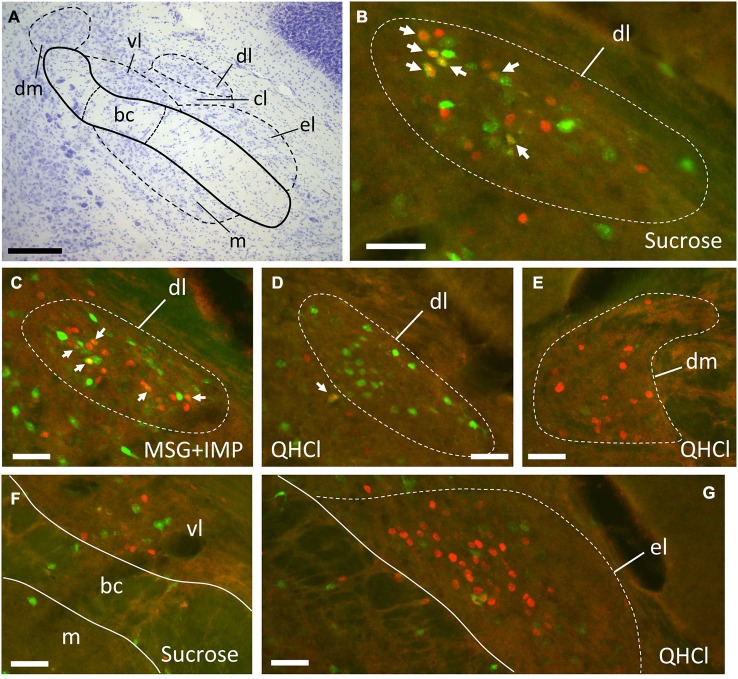
**Examples of FG and FLI labeling in the PbN according to location and stimulus. (A)** Nissl-stained section through intermediate level of gustatory PbN, showing delineation of subnuclei. **(B–G)** fluorescent labeling for FG (green) and FLI (red) in the ipsilateral PbN. **(B)** Intraoral sucrose evokes robust FLI expression in cells in the dl, a subnucleus that possesses a cluster of LH-projecting cells; several double-labeled cells can be seen (arrows). **(C)** The stimulus mixture MSG + IMP also evokes robust FLI in cells in this region, and double-labeled cells are present (arrows). **(D)** QHCl did not evoke FLI in cells in the dl. However, QHCl evoked robust FLI in cells in the dm **(E)** and el **(G)**, but with few double-labeled neurons. **(F)** Sucrose-evoked FLI and FG cells in the gustatory waist region of the PbN. Scale bars = 100 μm for **(A)**, 50 μm for all other images **(B–F)**.

Tracer injection sites in the LH were reconstructed for all mice and are shown in Figure 1. We next examined the distribution of FG, FLI, and double-labeled cells in PbN subnuclei across 6–7 sections per mouse. FG labeling was typically seen throughout the cell soma, including the nucleus, whereas FLI labeling was confined to the cell nucleus (Figure 2). FG labeled cells were found in every subnucleus examined, at all levels examined, but overall appeared in greater density in the lateral subnuclei, including a distinct and consistent cluster in the dorsal lateral (dl) subnucleus (Figure 3). FLI cells were distributed among all subnuclei, with stimulus-dependent expression in some areas such as the dorsal medial (dm) and dl subnuclei. Most double-labeled cells were found in the dl; few were seen in any other subnucleus (Figure 3).

**Figure 4:**
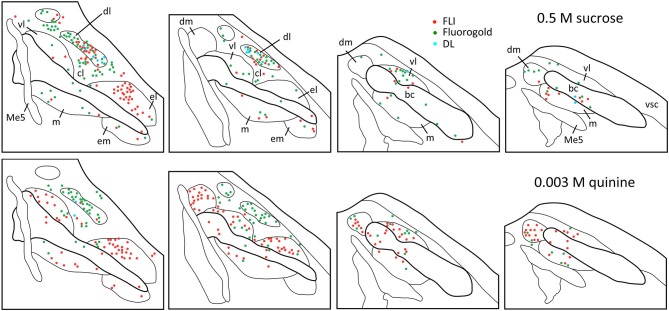
**Representative plots showing distributions of FG (green circles), FLI (red circles) and double-labeled (blue circles) neurons in the PbN in response to intraoral sucrose (top) and QHCl (bottom).** Sections are organized from rostral (left) to caudal (right), and spaced approximately 80 μm apart. Each set of plots was constructed from a single mouse. Me5, mesencephalic trigeminal nucleus; vsc, ventral spinocerebellar tract.

Plots of FG and FLI labeling in representative mice following stimulation with sucrose or quinine demonstrate the extent of labeling in PbN subnuclei across the rostral—caudal axis (Figure 4). These two stimuli were chosen for these plots as they evoked not only the greatest number of FLI cells, but also the greatest contrast in terms of variation in subnuclear label. Caudally, quinine evoked FLI in the “gustatory waist” region; this region includes the medial (m) and ventral lateral (vl) subnuclei, as well as in the dorsal brachium conjunctivum (bc). Quinine also elicited FLI in a large number of cells in the dm, along its rostral-caudal extent. Sucrose resulted in label in the gustatory waist, along with strong FLI expression in the dl. Both stimuli evoked FLI in the external lateral subnucleus (el).

**Figure 5:**
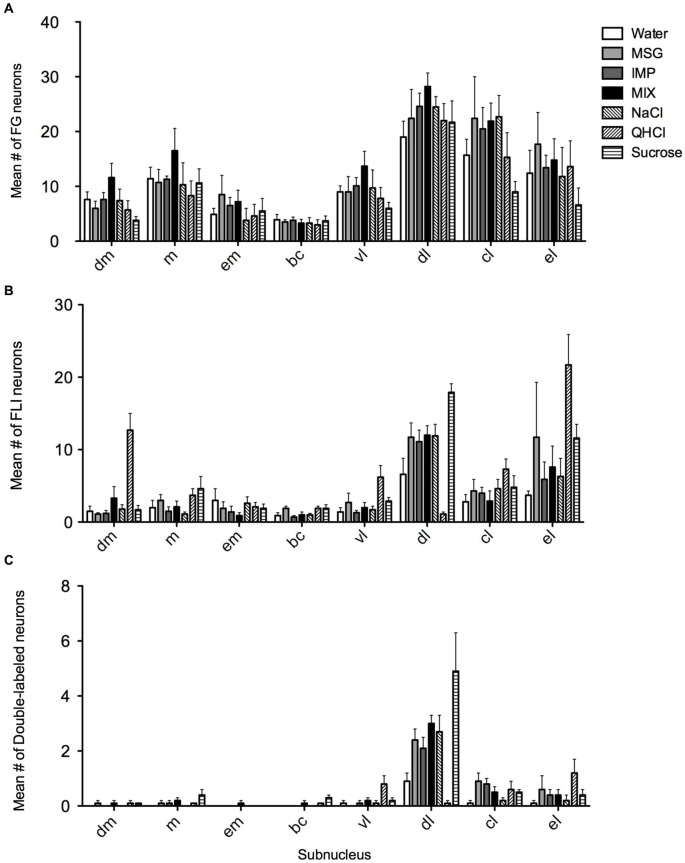
**Quantification of FG, FLI and double labeling in the PbN by subnucleus. (A)** Mean numbers of LH-projecting neurons varied according to subnucleus but not across stimulus groups. **(B)** Mean numbers of FLI neurons varied among stimuli in some subnuclei, as did double-labeled cells **(C)**.

FG and FLI cells, as well as double-labeled cells, were quantified within each subnucleus (mean count across sections) and averaged across mice for each stimulus (Figure 5). As expected, average counts of FG cells (Figure 5A) did not significantly vary with stimulus (*F*_(6,28)_ = 0.97, *p* = 0.46), although they varied significantly according to subnucleus (*F*_(7,196)_ = 61.9, *p* < 0.00001). There was not a significant stimulus vs. subnucleus interaction (*F*_(42,196)_ = 0.98, *p* = 0.51). When data were collapsed across stimulus and analyzed with a one-way ANOVA, *post-hoc* multiple comparisons tests revealed significant variation in FG counts among subnuclei (*F*_(7,272)_ = 35.8, *p* < 0.0001; see Table 1). In particular, significantly more FG cells were found in the dl than in any other subnucleus; more cells were also found in the cl than in other areas.

**Figure 6:**
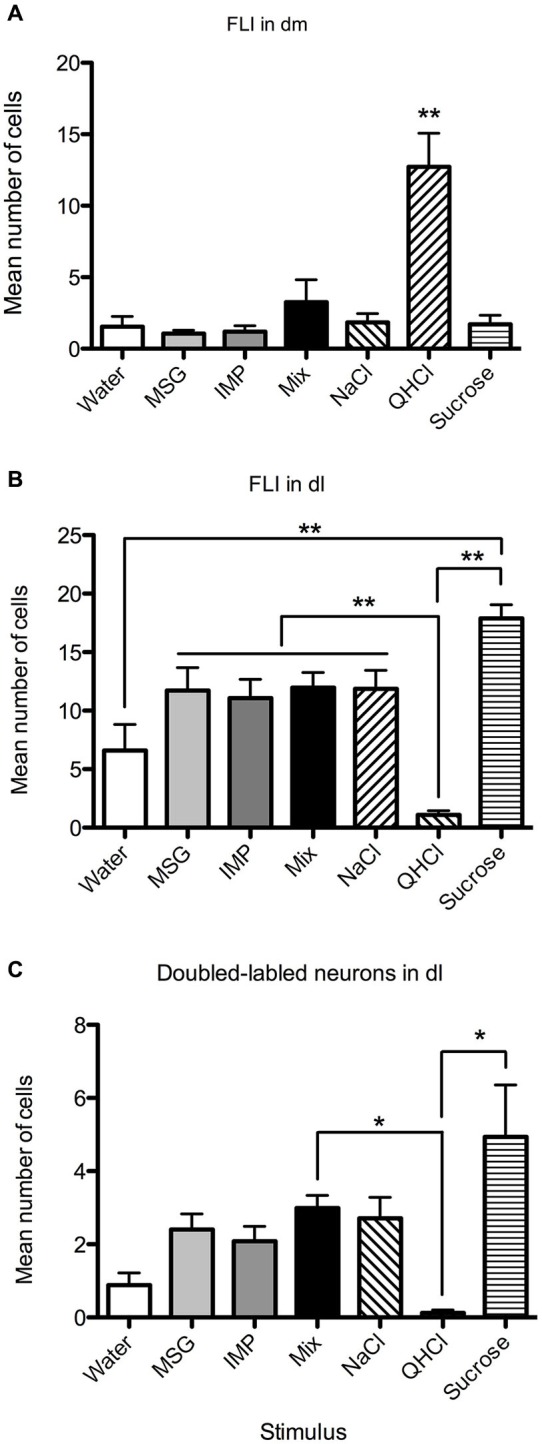
**Quantification of labeled neurons in particular PbN subnuclei.** These plots highlight group differences shown in Figure 5 (same data). **(A)** QHCl evoked significantly more FLI in cells in the dm than did other stimuli. **(B)** Sucrose evoked significantly more FLI in cells in the dl than QHCl or water; all groups significantly differed from quinine. **(C)** QHCl stimulation resulted in significantly fewer double-labeled cells than were found with sucrose or the mixture. Significance was established with *post-hoc* testing (**A–B**, Bonferroni; **C**, Dunn’s test); * *p* < 0.05; ** *p* < 0.01.

Overall, FLI counts did not vary significantly by stimulus (*F*_(6,28)_ = 2.20, *p* = 0.07), but did vary by subnucleus (*F*_(7,196)_ = 42.50, *p* < 0.00001). A significant interaction (*F*_(42,196)_ = 4.98, *p* < 0.00001) indicated stimulus variation in FLI was dependent on subnucleus. Counts were therefore examined within each subnucleus via one-way ANOVAs. Significant differences in FLI expression among stimuli were found within the dm, bc, vl, dl, and el (*F*s_(6,28)_ > 2.45, *p* < 0.05). Notably, quinine evoked FLI in more cells of the dm than did other taste stimuli (Figure 6A). Similarly, quinine also induced FLI in more cells in the vl than any stimulus except MSG or sucrose (Figure 5). In the dl, quinine elicited FLI in significantly fewer cells than any other stimulus (Figure 6B); sucrose also evoked more FLI expression than water.

Relatively few neurons in the PbN were double-labeled, and their distribution roughly matched that of FLI cells. A Kruskal-Wallis test revealed a significant difference between stimuli in the dl (*H* = 21.74; *p* < 0.01). *Post-hoc* tests indicate that there were significantly fewer double-labeled cells following quinine stimulation as opposed to sucrose or the mixture (Figure 6C). Differences in double-labeled cells were not simply a reflection of more FLI neurons in the sucrose group, as a higher proportion of these activated neurons co-expressed FG following sucrose (mean proportion ± SEM = 0.27 ± 0.06) as compared to quinine (0.07 ± 0.05) (Mann-Whitney test, *p* < 0.05).

## Discussion

### Distribution of labeled cells in the PbN

The number of neurons in the ipsilateral PbN retrogradely labeled from LH was (as expected) consistent across stimulus groups, but varied according to subnucleus, with the greatest expression tending to occur laterally, especially in the dl. This projection pattern is consistent with previous experiments in rats (Fulwiler and Saper, 1984), and counts were comparable with those in a recent paper that examined PbN neurons retrogradely labeled from forebrain targets, including LH, in C57BL/6J mice (Tokita et al., 2010).

The distribution of FLI in the mouse PbN varied according to stimulus, although this variation was largely driven by differential expression in three subnuclei: the dm, dl, and el. None of these areas is thought to have a pure gustatory function, instead being regions where there is overlap between gustatory and visceral input (Herbert et al., 1990; Karimnamazi et al., 2002). Intraoral (IO) stimulation results in stimulation of both gustatory oral receptors and postoral or visceral mechanisms, especially in the case of a highly preferred stimulus such as 0.5 M sucrose, which mice avidly consume. The mice likely even consume some small amounts of aversive stimuli such as 0.003 M quinine, a compound that the mice were observed actively rejecting during testing (although we did not quantify either intake amounts or taste reactivity behavior). Yamamoto and Sawa (2000a) directly compared IO and intragastric presentation of taste stimuli in rats in terms of FLI, including 0.5 M sucrose, 0.001 M quinine, and water. IO sucrose-evoked FLI was indistinguishable from that evoked by intragastric presentation in caudal, purely visceral areas of the nucleus of the solitary tract (NST), proving that IO administration results in a substantial post-ingestive footprint centrally. However, in areas where the IO stimulus evoked more FLI than intragastric presentation, a gustatory contribution could be inferred. In their study this included the rostral, gustatory NST, and in the PbN, the gustatory “waist” area (m, vl, and bc), as well as the dl (Yamamoto and Sawa, 2000a). Post-perfusion timing may also influence the relative activation of taste and visceral inputs. However, in a recent study (Hashimoto et al., 2009) robust FLI was observed in the dl of mice following intake of 0.1 M NaCl. This study used shorter post-perfusion times (30 min after stimulus onset) than the current study (2 h after stimulus onset), yet FLI patterns were similar to ours.

**Table 1:** **Mean counts of FG-labeled cells in each subnucleus (collapsed across stimulus groups)**.

**Subnucleus**	**Mean number of FLI cells (± SEM)**	**dm**	**m**	**em**	**bc**	**vl**	**dl**	**cl**	**el**
**dm**	7.10 ± 0.68							
**m**	11.34 ± 1.01	ns						
**em**	5.93 ± 0.80	ns	0.05					
**bc**	3.52 ± 0.28	ns	0.01	ns				
**vl**	9.33 ± 0.81	ns	ns	ns	0.01			
**dl**	23.19 ± 1.24	0.01	0.01	0.01	0.01	0.01		
**cl**	18.14 ± 1.70	0.01	0.01	0.01	0.01	0.01	0.05	
**el**	12.95 ± 1.53	0.01	ns	0.01	0.01	ns	0.01	0.05

*p-values from Bonferroni’s multiple comparison test*.

In mouse taste electrophysiological experiments, gustatory neurons are found clustered caudally in the waist region. More rostrally, taste neurons are found in the m, vl, and el (Tokita and Boughter, 2012; Tokita et al., 2012). In these studies, taste responses were measured in individual PbN neurons in response to oral stimulation; recordings were only made in clearly isolated cells (signal exceeded baseline activity by ≥ 2 SD). Taste-responsive neurons were not found in the dl or dm, although this could be due to a bias for recording close to the site of greatest taste-evoked activity (which is in the waist); moreover, cell bodies in the dl are small (Figure 2; see also Halsell and Frank, 1991), making electrophysiological isolation difficult. On the other hand, Halsell and Frank (1991) did not report multiunit taste activity in the dl in hamsters. Interestingly, we did not see many differences among stimuli in terms of FLI expression in the subnuclei comprising the waist area. An exception to this was quinine, which evoked FLI in significantly more cells in the vl than all stimuli except sucrose. Overall, our data indicated differential patterns of FLI in the PbN between sucrose and quinine, dependent on expression differences in a few subnuclei. The tendency for topographical segregation between taste qualities, especially sweet and bitter stimuli, has been previously noted in the gustatory brainstem (NST or PbN) in physiological or anatomical studies (Yamamoto et al., 1994; Harrer and Travers, 1996; Sugita and Shiba, 2005; Tokita and Boughter, 2012; Tokita et al., 2012).

Another notable finding in the current study was the apparent absence of synergistic umami effects in the PbN. In neurophysiological studies, mixtures of glutamic acid salts (such as MSG) and 5’ -ribonucleotides (such as IMP) produce a response that is much greater than the sum of the individual components (e.g., Adachi and Aoyama, 1991; Ninomiya et al., 1992; Sako and Yamamoto, 1999; Sako et al., 2003). In the mouse PbN, 0.1 M monopotassium glutamate (MPG) mixed with 0.01 M IMP produced a synergistic neural response that was more or less equivalent to that evoked by 0.5 M sucrose (Tokita and Boughter, 2012; Tokita et al., 2012). In the current experiment, the 0.1 M MSG + 0.1 M IMP mixture evoked FLI in roughly the same numbers of cells as either MSG or IMP alone, and far <0.5 M sucrose. It is quite possible that synergism is not particularly evident with the FLI technique; i.e., effects are only seen physiologically in terms of variable response within an individual neuron. The relative strength of a response is not readily evident within a single FLI neuron, only the fact that the stimulus activated it or not. This may also help explain the relative lack of a greater FLI response evoked by all of the taste stimuli relative to water. The lack of synergism may also reflect that many of the FLI neurons are responding to post-ingestive rather than orosensory stimulation. Moreover, studies have demonstrated that evoked physiological activity and FLI expression in any one population of neurons is not always correlated (e.g., Fenelon et al., 1993). In the gustatory brainstem, Travers (2002) showed that IO 0.3 M NaCl evoked little FLI in the NST in rats, even though this concentration is extremely potent in terms of evoking neurophysiological response in neurons in this area (e.g., Giza and Scott, 1991). Hence, the functional interpretation of activity in any one region or subnucleus may vary depending on technique.

### Activation of the LH projection

Few activated projection (double-labeled) cells were found in most PbN subnuclei. Small numbers were found in the cl and el, but the largest concentration was found in the dl. This was not merely a consequence of larger numbers of labeled cells (either FG or FLI), as the amount of double-labeled cells in the dl was several-fold higher than in the el, despite robust labeled cell counts in the latter region (e.g., Figure 5). Across stimuli, double-labeled cells in the dl did closely reflect the number of FLI cells. Sucrose stimulation resulted in the greatest number of activated LH-projecting neurons, followed by roughly equivalent numbers for MSG, IMP, the MSG + IMP mixture and NaCl. Water was slightly less effective than these stimuli, and quinine stimulation resulted in almost no double-labeled cells.

Based on this evidence, it is reasonable to draw one of two conclusions: the first is that the dl, and its projection to the LH, is primarily concerned with visceral stimulation. Sucrose might evoke a stronger FLI response in this region as opposed to other stimuli due to post-ingestive chemosensory/nutrient signaling (e.g., Kokrashvili et al., 2009; Fernstrom et al., 2012). On the other hand, visceral stimulation might correspond to gastric loading—IO sucrose likely results in the largest amount of ingested fluid. In these scenarios quinine may not be an effective post-oral stimulus, as mice reject it. In rats, Yamamoto and Sawa (2000a, b) found that intragastric delivery of quinine does not elicit FLI in the dl in rats; moreover, IO saccharin or sucrose elicited substantially more FLI in this region than was found following intragastric delivery. These results argue against a purely visceral interpretation of our data.

A second possibility is that IO-evoked activity in the dl is more concerned with gustatory-related appetitive behavior. Indeed, Yamamoto et al. (1994) characterized the dl (in rats) as an “ingestive” area due to the fact that appetitive or hedonically positive or neutral taste stimuli such as sucrose, saccharin, and NaCl, but not aversive stimuli like quinine or HCl, evoke a characteristic cluster of FLI neurons in this subnucleus. This pattern was found with either IO infusion or voluntary drinking (Yamamoto et al., 1994; Yamamoto and Sawa, 2000a). The use of IO infusion in our study limits inference of palatability of particular stimuli (especially those other than sucrose) as opposed to measuring spontaneous consumption. However, our FLI data adhere to this previously described pattern, and extend it by showing that some percentage of these activated cells project directly to the LH. As mentioned above, potential sensory input to the LH from the PbN in this subnucleus may reflect a combination of both gustatory and post-ingestive factors.

PbN afferents to the LH include dense terminal labeling at tuberal levels (Norgren, 1976), where there are also clusters of orexin- (ORX) and melanin concentrating hormone- (MCH) expressing neurons (Broberger et al., 1998). Both of these neuropeptides have been implicated in feeding behavior as orexigenic (appetite-producing) agents, especially involved in the intake of palatable foods such as fats or sugars (e.g., Barson et al., 2013). Niu et al. (2010) recently showed that lateral PbN neurons make excitatory synaptic contacts with ORX neurons in the LH. In a recent study, Li et al. (2013) examined physiological responses in LH neurons to taste stimuli in awake and behaving rats. Gustatory-responsive neurons could be divided into either palatable-preferring or aversive-preferring subtypes; physiological evidence suggested that palatable-preferring neurons are clustered near, and may in fact actually be, ORX or MCH neurons. Our data are consistent with their model, showing that palatable taste compounds selectively activate lateral PbN afferents to the LH. There may exist a feedback loop, as LH ORX and MCH neurons also project back to PbN, among other targets (Moga et al., 1990; Touzani et al., 1993; Peyron et al., 1998; Cutler et al., 1999).

## Conclusion

Our work demonstrates that stimuli that have been shown to be behaviorally preferred by mice, including certain sweet and umami stimuli, activate a subset of neurons in the PbN that project to the LH. This projection may be important in terms of direct taste- and intake-related input to LH neurons that may initiate, maintain, and contribute to control of feeding. Further work is necessary to show how neural activity in LH neurons may be specifically modulated by PbN input.

## Author contributions

Conceived and designed the experiments: Kenichi Tokita, William E. Armstrong, Steven J. St. John and John D. Boughter. Performed the experiments: Kenichi Tokita. Analyzed the data: Kenichi Tokita, William E. Armstrong and John D. Boughter. Wrote the paper: Kenichi Tokita, Steven J. St. John, William E. Armstrong and John D. Boughter.

## Conflict of interest statement

The authors declare that the research was conducted in the absence of any commercial or financial relationships that could be construed as a potential conflict of interest.
